# Supplementary material for: Novel Driver Strength Index highlights important cancer genes in TCGA PanCanAtlas patients
Source: PeerJ. 2022 Aug 11;10:e13860. doi: 10.7717/peerj.13860 (PMC9375969; doi:10.7717/peerj.13860)
Supplement: Supplemental Information 2 [file peerj-10-13860-s002.zip › COHORTS/genes plots/cohorts/2021_8_16_14_9_distribution_cohorts_PANCAN.pdf]

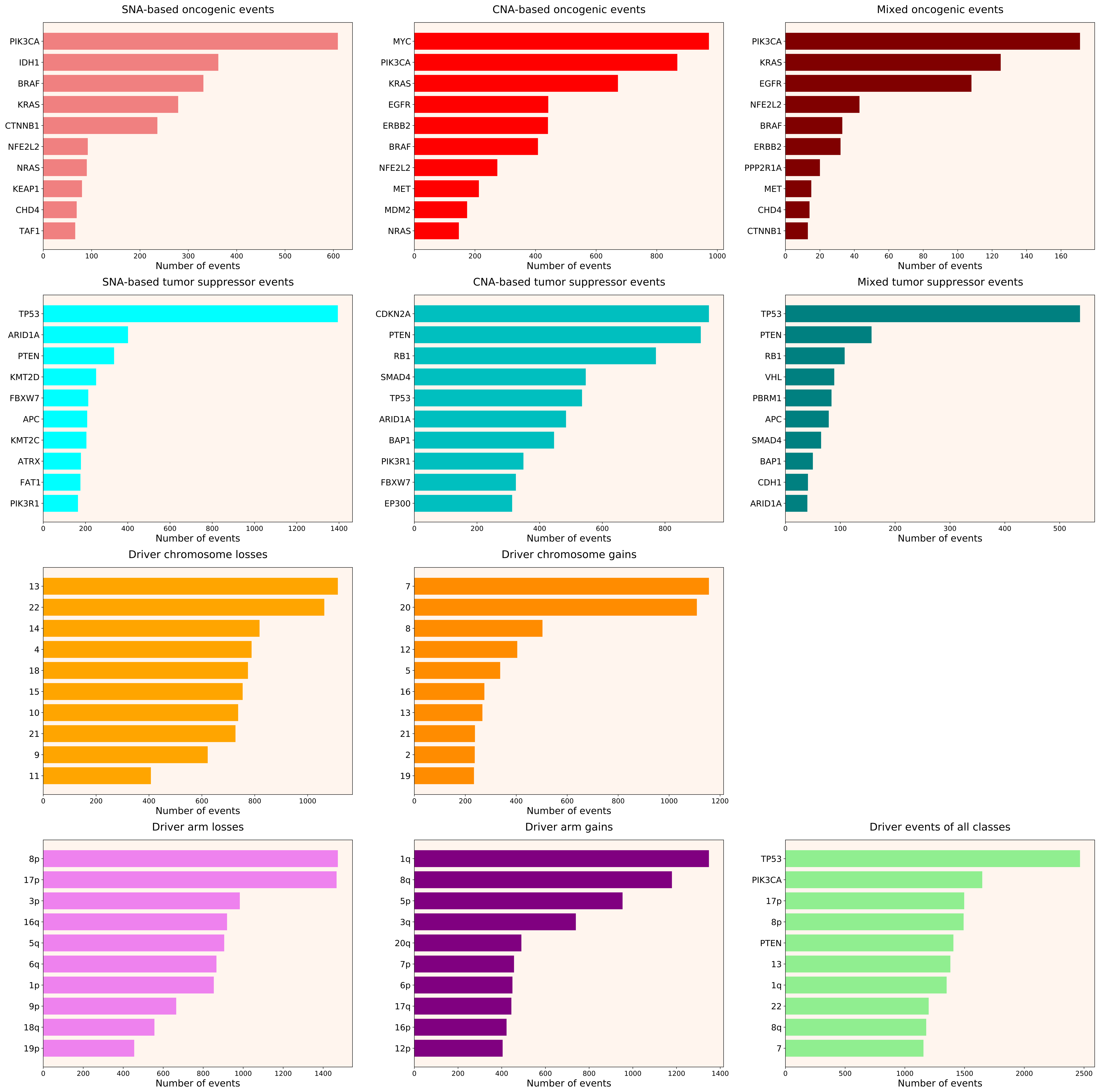
SNA-based oncogenic events
PIK3CA
IDH1
BRAF
KRAS
CTNNB1
NFE2L2
NRAS
KEAP1
CHD4
TAF1
0 100 200 300 400 500 600
Number of events
CNA-based oncogenic events
MYC
PIK3CA
KRAS
EGFR
ERBB2
BRAF
NFE2L2
MET
MDM2
NRAS
0 200 400 600 800 1000
Number of events
Mixed oncogenic events
PIK3CA
KRAS
EGFR
NFE2L2
BRAF
ERBB2
PPP2R1A
MET
CHD4
CTNNB1
0 20 40 60 80 100 120 140 160
Number of events
SNA-based tumor suppressor events
TP53
ARID1A
PTEN
KMT2D
FBXW7
APC
KMT2C
ATRX
FAT1
PIK3R1
0 200 400 600 800 1000 1200 1400
Number of events
CNA-based tumor suppressor events
CDKN2A
PTEN
RB1
SMAD4
TP53
ARID1A
BAP1
PIK3R1
FBXW7
EP300
0 200 400 600 800
Number of events
Mixed tumor suppressor events
TP53
PTEN
RB1
VHL
PBRM1
APC
SMAD4
BAP1
CDH1
ARID1A
0 100 200 300 400 500
Number of events
Driver chromosome losses
13
22
14
4
18
15
10
21
9
11
0 200 400 600 800 1000
Number of events
Driver chromosome gains
7
20
8
12
5
16
13
21
2
19
0 200 400 600 800 1000 1200
Number of events
Driver arm losses
8p
17p
3p
16q
5q
6q
1p
9p
18q
19p
0 200 400 600 800 1000 1200 1400
Number of events
Driver arm gains
1q
8q
5p
3q
20q
7p
6p
17q
16p
12p
0 200 400 600 800 1000 1200 1400
Number of events
Driver events of all classes
TP53
PIK3CA
17p
8p
PTEN
13
1q
22
8q
7
0 500 1000 1500 2000 2500
Number of events
